# Mothers’ experiences and perceptions about care provided during home deliveries in Alwa sub county, Kaberamaido district, Uganda– a qualitative study

**DOI:** 10.3389/fpubh.2023.1180945

**Published:** 2023-10-10

**Authors:** Benbella Dektar, Anita Normans Beckford, Jonny Kemba, Belinda Crayson

**Affiliations:** ^1^School of Public Health, College of Health Sciences, Makerere University, Kampala, Uganda; ^2^Africa Centres for Disease Control and Prevention, Lusaka, Zambia; ^3^Save the Children International, Milton Keynes, United Kingdom; ^4^Global Quest Consulting Limited, Kampala, Uganda; ^5^Coffey International, Sydney, Australia

**Keywords:** mothers’ experiences and perceptions, home deliveries/home birth, maternal care, traditional birth attendants (TBA), Kaberamaido district, Uganda (sub Saharan Africa), descriptive qualitative research design

## Abstract

**Introduction:**

In Uganda 27% of deliveries take place outside a health facility. The existing gaps in quality of maternal and newborn health care must be addressed for Uganda to attain its health targets and consequently its economic targets. Some of the gaps include but are not limited to; ill-equipped healthcare facilities in rural settings, inadequate client/customer care skills by healthcare providers, and health worker absenteeism especially in the night hours. In Kaberamaido District, only 38.3% of the deliveries in Alwa sub county took place at a health facility. Despite the district local government and stakeholder efforts to promote health facility-based deliveries, sadly, a very low proportion of women use the health facilities for delivery. We sought to explore mothers’ experiences and perceptions about care provided during home deliveries in Alwa sub county, Kaberamaido district.

**Methods:**

The study adopted a cross-sectional descriptive qualitative design. We purposively included 115 mothers who delivered outside the health facility and consented to participate and those who lost their babies within the last 24 months preceding the study. Other participants included in the study were village health team (VHT), traditional birth attendants (TBA) and older women. The main instruments used to collect data were focus group discussion (FGD) and in-depth interview (IDI) guides. All voice recordings from FGDs and interviews were transcribed and translated from the local language (Kumam) into English. Thematic content analysis was used to synthesize data by applying codes to segments of the transcripts upon which major domains were derived. Key findings were synthesized and quotes were carefully selected based on their relevance and representativeness to the analysis and study objective.

**Results:**

Our findings indicated general satisfaction with the care provided during and after home delivery by TBAs as expressed by mothers. Motivation to seek services from TBAs was attributable to their vast experience spanning decades with history of safe delivery. Few mothers expressed discontent with TBA services citing abuse and rudeness.

**Discussion:**

Our study underscored the common view that TBAs effectively managed home deliveries, providing satisfactory care to mothers. However, concerns emerged on TBAs’ capacity to manage complications, emphasizing a need for their reintegration into formal healthcare systems, alongside further training, and standardization in maternal care practices.

## Introduction

Globally, approximately 830 women die every day from preventable causes related to pregnancy and childbirth, with over 99% occurring in low-resource countries (1).

In Uganda, maternal and perinatal death reviews (2) and population situation analysis reports (3) indicate that quality of care is to blame for the country’s failure to meet maternal mortality reduction targets. In 2011, the maternal mortality ratio (MMR) in Uganda was 438 deaths per 100,000 live births. Despite the reduction to 336 deaths per 100,000 live births, the country still failed to attain its Millennium Development Goal target of 131 per 100,000 live births by 2015 (4). About 27% of deliveries in Uganda take place outside a health facility (4). For Uganda to attain its health targets and consequently its economic targets, the existing gaps in quality of maternal and newborn health care must be addressed (5). Some of the gaps in the quality of maternal and newborn health care include but are not limited to; ill-equipped healthcare facilities in rural settings, inadequate client/customer care skills by healthcare providers, and health worker absenteeism especially in the night hours.

Improving quality of care for women requires an understanding of their cultural values, previous experiences, and perceptions of the role of the health system (6). In addition, the 2013 maternal and perinatal death review report reveals that delayed care-seeking was an important avoidable factor that contributed to 56% of maternal deaths (7). Although meeting the needs and expectations of women is an important part of client-centered care, client satisfaction in health facilities in Uganda is low (8) hence one of the major drivers of home deliveries. Research demonstrates that there is a complex interplay of experiences of mistreatment and lack of support that impact women’s childbirth experiences and outcomes (9, 10). This implies that once birth outcomes are good, mothers are more likely to continue seeking health facility care but might be discouraged in the event of adverse birth outcomes. Whereas peer influence/community perception plays a role in a mother’s decision on where to delivery from, it was not a major factor identified in this study and therefore not of great significance even though some communities believed that TBAs had enough midwifery knowledge, skills, and that they were trustworthy. It is therefore worth noting that, mothers’ experiences and perceptions about care provided during home birth largely depends on the outcome of birth.

The Kaberamaido district health management information system report 2016 indicated that only 38.3% of the deliveries in Alwa sub county took place at a health facility (10). This implies that the majority of deliveries, which account for 61.7% took place at home, likely under the assistance of medically unskilled birth attendants. Despite the district local government and stakeholder efforts to promote health facility-based deliveries such as; routine sensitization campaigns on the merits of health facility-based deliveries, equipping facilities with necessary equipment, medicines, and stocking essential therapeutics and diagnostics to facilitate delivery, sadly, a very low proportion of women use the health facilities for delivery.

Numerous studies suggest that the reasons for health facility delivery include accessibility, good perceptions about the safety of health facility births, positive attitudes toward health providers and quality of care received for facility births (11–13). Conversely, reasons for homebirths include the cost-effectiveness and convenience of homebirths, having family members nearby during women’s labor experiences, and maintaining traditional childbirth and postpartum practices (14, 15). Other reasons for homebirths include social pressure from family members, social norms, and past experiences of using TBAs (15, 16). A study conducted in Zambia revealed that women preferred services of the TBAs because they were available and easily accessible, affordable and pragmatic. Moreover, the community believed that TBAs had enough midwifery knowledge, skills, and that they were trustworthy (17).

Therefore, our study aimed at exploring mothers’ experiences and perceptions about care provided during home deliveries in Alwa sub county, Kaberamaido district located in Eastern Uganda. The results of the study provide a benchmark and recommendations on how best to sensitize pregnant mothers and their spouses about the demerits of home-based deliveries with an ultimate goal of eliminating the problem. In addition, our findings provide information on how district-based policy-makers and healthcare workers can reinforce facility-based deliveries and mitigate factors influencing home deliveries. Such information can be used to design byelaws and ordinances at district level with the aim of eliminating home-based deliveries.

## Materials and methods

### Study site

We conducted the study in Alwa sub county, Kaberamaido district located in rural Eastern Uganda. The district headquarters are situated in Kaberamaido Town Council, approximately 434 km from Kampala City; the capital of the Republic of Uganda. The district lies approximately between latitudes 10 33’N – 20 23’N and Longitudes 300 01′ E - 340 18′E. The district has a population of 215,026 people with a population growth rate estimated at 3.3% per year (18). The main local language spoken in the district is Kumam.

### Study design

The study adopted a community-based, cross sectional descriptive qualitative research design. The entire study was conducted from August 2020 to February 2021.

Given that the study sought to explore mothers’ experiences and perceptions about care provided during home deliveries, a cross sectional descriptive qualitative design was an absolute imperative. This design prompts discussion thereby enabling solicitation of experiences, perceptions and opinions which can be documented in form of verbal narrations.

### Study population

We purposively selected 115 mothers who delivered outside the health facility and consented to participate as well as those who lost their babies within the last 24 months preceding the study in order to minimize recall bias. We implemented a purposive sampling strategy to include the 115 mothers due to the specificity of our research. This enabled us to target participants with unique experiences. Leveraging their familiarity with the childbearing women within their respective villages, the Village Health Team (VHT) members, traditional birth attendants, and local leaders played a pivotal role in identifying potential study participants and facilitating their enrolment into the study.

Upon meeting the criteria, mothers were invited to partake in the study. Throughout the process, we strictly adhered to ethical guidelines, focusing on informed consent and confidentiality. Despite potential concerns about representativeness of non-probability sampling techniques such as purposive sampling, our objective was to gain in-depth insights into home birthing experiences. The reason for their inclusion was to help determine whether the deaths were associated with home deliveries as a result of the inadequate skills of the TBAs by ascertaining the conditions (circumstances) that led to the babies’ death. This group particularly included women who delivered at home and lost a child at delivery or during the neonatal period for the reasons stated above. Whereas this was not a comparative study, we note that there were no compelling differences between mothers whose babies survived and their counterparts who delivered at home and lost their babies.

To ensure that the right study participants were recruited, the VHTs were carefully briefed about the category of participants and inclusion/exclusion criteria. Study participants were approached face-to-face. The VHTs physically reached the mothers prior to our arrival and scheduled appointments for the interviews and focus group discussions which we held at the homes of the participants.

Our study employed a multi-method qualitative approach, encompassing focus group discussions (FGD), key informant interviews (KIIs), and in-depth interviews (IDIs). This amalgamated approach was fundamental as it allowed us to grasp the heterogeneous perspectives of the various respondents who graciously consented to participate in our research. We conducted nine focus group discussions. Each FGD incorporated seven to twelve participants, a count carefully calibrated to maintain an equilibrium of experiences and viewpoints. Our nine FGDs were meticulously curated to include a spectrum of mothers who had given birth outside health facilities and those who suffered neonatal loss in the last 24 months postpartum. The composition, influenced by participant availability and willingness, encouraged diverse discussions. While some groups had a higher ratio of mothers experiencing neonatal loss, others had more successful home deliveries. This approach minimized bias, promoting nuanced understanding of home deliveries and neonatal mortality. Due to ethical constraints and participant confidentiality, exact group compositions cannot be disclosed. However, careful planning was involved to yield rich, varied, and dependable data. We also conducted 29 in-depth interviews. To obtain supplementary data, 12 key informant interviews (KII) were conducted. We note that saturation points upon which no new information was being generated were reached across all interviews and discussions held with study participants. Key informants included Village Health Team (VHT) members, Traditional Birth Attendants (TBA) and older women.

### Data collection instruments and methods

Data was collected between September and October, 2020. In-depth interview and focus group discussion guides were the main data collection instruments used. All the instruments (IDI guides, FGD guides, and KII guides) including the questions and prompts used for data collection during the study were not adopted from earlier studies but developed and provided by the investigators. The instruments were developed in English and translated to the local language (Kumam), they were later back-translated to English to guarantee consistency in content. They comprised of open-ended questions meant to fortify discussions.

We carefully selected three local research assistants, (two graduates and one Advanced level leaver) fluent in the English and local language (Kumam) who elicited responses from the study participants by administering the data collection instruments. We opted for research assistants without medical background so as to minimize social desirability bias. They were trained by the Principal Investigator (PI) for two days. The training focused on the study objective, data collection techniques and ethical considerations. We pre-tested all the data collection instruments so as to assure internal consistency and validity, ensuring that no discrepancies and ambiguity existed in the instruments. Pre-testing was done in Gwetom village located in Kaberamaido Parish, Kaberamaido sub county, with women who delivered outside the health facility in the past 24 months. No major ambiguities were identified and neither were major revisions required, however, we noticed the need to paraphrase and probe deeply so as to elicit detailed responses.

In-depth interviews lasted an average of 47 minutes while FGDs lasted an average of 60 minutes.

### Data management and analysis

All audio recordings from FGDs and interviews were transcribed word by word and translated from the local language (Kumam) into English by research assistants. Unclear terms discovered during the interviews were discussed and clarified before commencement of the transcriptions. Field notes were taken during the interviews and FGDs and were enriched by the transcriptions of the audio recordings. All data collected were edited to clearly identify the trends in the responses. The principal investigator (PI) read through all transcripts from the FGD and IDI guides with major focus on identifying comments and narrations that were striking and of interest in line with the study objective.

Analysis of the data was guided by the consolidated criteria for reporting qualitative studies (COREQ) which recommends a 32-item checklist for interviews and focus groups (19).

To ensure reliability of the data, we deployed intercoder reliability involving two independent analysts with quantitative data coding skills who agreed on the coding of the content of interest with an application of the same coding scheme. The codes developed by the independent analysts were compared with the codes produced by the investigators. Codes with commonalities identified were used. The data were manually coded.

The data were first coded to identify the overarching ideas, thoughts, and opinions of the study participants. It is from the codes that memos were derived given that participant narrations of their experiences and perceptions were often long. Our study relied more on the memos since we needed to quote verbatim to help back our claims hence the main reason for manual coding as opposed to use of computer software. The striking narrations were written down on the sides of the transcripts as memos. It is from the similar and common narrations which emerged from the memos that major domains and themes were derived. We then synthesized the key findings and carefully selected quotes based on their relevance and representativeness to the aim of the analysis. To protect the identity of the study subjects, we used their ages as reference where verbatim was quoted.

As part of accountability and disclosure, we organized four community feedback sessions where we disseminated our findings in the simplest forms possible to the study participants and community members. We also held a validation workshop at the District Health Office where we disseminated our findings from a general and technical point of view.

### Ethics approval and consent to participate

Whereas our study was not a clinical trial or experimental in nature, it went through a rigorous review by the Makerere University Research and Ethics Committee which granted the approval for the study to be conducted.

Further still, to ensure that prospective participants made informed choices regarding whether or not to participate in the study, the investigators and research assistants comprehensively explained the purpose, objective, risks and benefits of the study, and how data would be collected from each prospective participant in the local dialect they best understood.

In the course of this study, informed consent was elicited orally from the participants, a majority (62%) of whom exhibited illiteracy. To substantiate their verbal consent, study participants authenticated their verbal agreement by imprinting their thumb on the consent form. The confidentiality of the participants was meticulously preserved throughout the data collection, analysis, and dissemination phases, thereby upholding the ethical standards of research.

## Results

### Socio-demographic characteristics of study participants

Four of the 115 mothers (3.5%) lost their babies either during delivery or during the neonatal period. Their characteristics are summarized in Table 1. The majority (41%) were aged 25–34 years, and 33% were aged 17–24 years while 26% were over 35 years of age. In terms of education, many (62%) of the mothers reported having not attained any formal schooling while 29% had attained primary school education and few (10%) reportedly attended secondary school. The majority (32%) had parity of 2–4 while 80% of the mothers reported depending on farming for their basic needs—hence peasants.

**Table 1:** Socio-demographic characteristics of study participants (*N* = 115).

Variable	Frequency, *N*	Percentage, %
*Age group*		
17–24 years	38	33%
25–34 year	47	41%
35+	30	26%
*Education level*		
Primary	33	29%
Secondary	11	10%
None	71	62%
*Parity*		
1	29	25%
2–4	37	32%
5+	18	16%
*Occupation*		
Peasant	92	80%
Hairdresser	14	12%
Retail business	09	8%

There were mixed perceptions about the maternal care provided by Traditional Birth Attendants (TBAs) during and after deliveries. Table 2 reveals the essential factors which contributed to quality maternal care. These factors included TBAs’ skills, positive perceptions about sanitation, positive attitudes during delivery, and quality postnatal care. These insights underscore the significance of TBAs’ roles in maternal health in the study area.

**Table 2:** Key factors in maternal care provided by traditional birth attendants, *N* = 115.

Factors in maternal care provided by TBAs	N	%
TBAs were skilled for delivery and handling complications	18	15.7%
Positive perceptions about cleanliness at the birthplace	34	29.6%
Positive perception and attitudes by TBA during delivery	52	45.2%
Good postnatal care by TBA	11	9.6%
Total	115	100%

### Perceptions about skills of TBA in delivering and handling complications

Our study revealed mixed feelings among respondents regarding their perceptions and experiences with care provided during home delivery, with TBAs being assessed both positively and negatively. Results indicated that some mothers preferred TBAs over health facility staff because they were satisfied with the services offered by the TBAs and had never encountered any complications for themselves and their babies after being delivered by TBAs. Whereas the common view was that TBAs were skilled and effectively managed deliveries with their associated complications if they arose, there were variations in how the different participants perceived TBAs, citing ineffectiveness by TBAs in managing complications such as retained placentae. One of the FGD participants said:

“She is a very kind old woman. Even when you go into labor and you do not have a mama kit, she is ever ready and keeps materials required to deliver women in her home especially the polyethene paper, surgical blade, cotton and threads. Women in this village prefer her over health facility staff because the care she provides is better than the care you get at the health facility.” (20-year-old mother).

From this narration, we noticed the perceived skill and experience possessed by the TBAs in handling complications as opposed to health workers.

On the other hand, however, our study revealed that some mothers were discontented with the care provided during home delivery regardless of whether the birth attendant was trained, skilled and experienced. To confirm this, one of the IDI respondents shared her sentiments, saying:

“I do not prefer TBAs, I only go to the TBA when I have no option. I suffered a retained placenta for nearly two days and my baby’s umbilical cord swelled and bled. The person who delivered me was medically unskilled and delivers women in an unhygienic hut which is dusty and congested. After delivery, I had to go to the health facility for better care”, (37-year-old mother).

Neonatal respiratory distress, one of the common birth complications reported by the study participants results from difficulty/failure of a newborn to breath normally soon after delivery. Failure to recognize this on time can often be fatal. A 19-year-old mother has to bear the pain that comes along with the loss of a newborn baby. It is unimaginable the magnitude of trauma and psychological anguish she had to endure more so at her teen age as she narrated her story: *“I was helped to deliver by a traditional birth attendant at home. I had an abrupt onset of labor at 3:00 am in the night and experienced rapid contractions, I delayed to push as I waited for my husband who attempted to find a motorcycle to rush me to the health facility. The nearest rider he found hesitated because most of them do not accept to operate late at night for security reasons. We were left with no option but to seek the services of the nearby TBA who has successfully helped many mothers in my neighborhood. Unfortunately, my baby died soon after delivery.”* She explained. *“In fact, much as she’s skilled and experienced, I was not contented and happy with how she went about the entire delivery as she assisted me.”* She added. Under such appalling circumstances, we probed this young 19-year-old mother further to try and establish what could have caused the death of her baby. In a sobbing tone she said that *“I was told by a nurse who arrived at home an hour later that my baby got tired, was not breathing well after delivery and needed to be medically resuscitated quickly.”* Whereas this young mother and her spouse were interested in having their baby safely delivered in a health facility, unfortunately, they had to rely on the TBA who herself did not have medical equipment to help save their baby.

### Perceptions about cleanliness of the delivery place used by birth attendant

Study participants were satisfied with the hygiene of the environments upon which the TBAs conducted their deliveries. For purposes of the study, a hygienic environment was known to be one which was dust-free, free of hanging cob-webs, chicken droppings and free of cracks/crevices harboring insects such as cockroaches and other arthropods. A 24-year-old mother reported that:

“… I did not have any problem with the place where she helped me deliver, she took me to a hut but it was clean, well swept and smeared with cow dung so there was no dust, not even cobwebs.”

In addition, a 27-year-old mother’s sentiments matched the above 24-year-old’s—reporting that:

“… I did not have any problem with the surroundings where she delivered me from. The environment was clean” (27-year-old mother).

These narrations from the respondents suggested their satisfaction with the cleanliness and hygiene of the surroundings where they delivered.

Comparatively, whereas there was general satisfaction with the surrounding hygiene, a 32-year-old study participant expressed dissatisfaction with the location where she was delivered. She had this to say:

“I was helped deliver by my grandmother. She took me to her bath shelter saying that she was afraid I would bleed a lot and did not want my blood to stain her house. It was dirty and smelly with a lot of algae and stagnant water. Luckily, I did not pick up any infection.”

This expression of discontent suggests that not all mothers approved of the surroundings within which they were helped to deliver by the birth attendants.

### Perceptions about conduct and attitudes of birth attendants during delivery

We established that respondents who were delivered by TBAs reported that they were warmly welcomed and received when they went for delivery. To affirm this, one FGD participant said:

“… I was well received and given a bed by the TBA when I arrived, she examined my cervix and told me that it was not yet fully open and that I should wait for about 10 minutes. She told me not to worry and that everything would be okay. She kept counseling me as we waited for the cervix to fully dilate”. (32-year-old mother).

Whereas the majority expressed that the conduct and attitude of TBAs was satisfactory, a few (minority) mothers said that the conduct and attitude of the TBAs was not satisfactory citing insults, abuse, carelessness and rudeness while conducting delivery. Evidence about this was reported by a 23-year-old mother who said that:

“She was very abusive and rude, at one point she left me alone in the house when I was in labor pains. She eventually helped me successfully deliver my baby but I was not happy with her attitude and rudeness.”

### Perceptions about postnatal care received

Participants reported being cared for during the post-natal period (at least for six weeks). They reported that those who delivered them massaged them with warm water at least twice a day. A 34-year-old mother affirmed this, saying that:

“… when she helped me to deliver, she asked me to stay with her for two days as she monitors my situation. She kept massaging me every day. Even when I want back home, she kept coming to massage me twice daily until I felt better and was strong enough to continue massaging myself without help.” (34-year-old FGD participant).

Another 26-year-old mother reported that:

“The woman who helped me deliver stays 11 km away, she is very experienced and I heard that she has been doing this for over 30 years now. I was very happy with the way she worked on me and care she offered me even after delivery.” “She even called me two days ago to find out how I was doing.” She went on.

The above narratives suggest women’s satisfaction with care provided by the birth attendants during and after birth.

Another IDI respondent shared similar sentiments, reporting that:

“My aunt helped to deliver but was also very helpful after the deliver, she kept checking on me every day and also kept massaging me for about three weeks. She also kept advising me that should I have any problem or feel uneasy, I should send someone to call her immediately so that she sees what to do or take me to the health facility in case of a serious complication after delivery”, (19-year-old IDI respondent).

Whereas immunization services were not offered by TBAs and other birth attendants during the post-natal period possibly due to lack of vaccines, with the above excerpts depicting satisfaction with care after delivery, our study revealed majority of the mothers expressed satisfaction with care provided by birth attendants during the postnatal period.

## Discussion

The results of this study regarding Mothers’ experiences and perceptions about care provided during home deliveries mirror the mixed findings found in the broader literature on this topic. In many resource-limited settings, TBAs play a critical role in providing maternal and newborn care due to factors such as limited access to healthcare facilities, cultural beliefs, and financial constraints (20). Our study noted mixed feelings among mothers on their perceptions and experiences with care provided during home delivery. Conversely, we noted that the majority of the mothers reported satisfaction with care provided by the TBAs during home deliveries as well as the postnatal period. This finding is consistent with studies conducted by (9, 10) which demonstrated that there is a complex interplay of experiences of mistreatment and lack of support that impact women’s childbirth experiences and outcomes. Some mothers in our study expressed satisfaction with the care provided by TBAs which echoes similar findings in other studies where TBAs were valued for their cultural appropriateness, familiarity, and supportive care. The satisfaction with the care provided by TBAs during delivery and the postnatal period is further linked to their clean history of delivery especially if they have handled deliveries over the years with no record of maternal death. This agrees with (17) who revealed that the community believed that TBAs had enough midwifery knowledge, skills, and that they were trustworthy. Moreover, they were available and easily accessible, affordable and pragmatic.

However, other participants in our study shared concerns about the capacity of TBAs to manage complications. This mirrors the sentiments in the literature that argue against relying solely on TBAs for deliveries due to their limited formal training in handling complications such as retained placentae (21). Such discrepancies in experiences and perceptions highlight the need for standardization and possible integration of TBAs into the formal health system, ensuring that they receive appropriate training to handle complications (22).

A significant portion of the literature has highlighted the essential role TBAs play in maternal healthcare, especially in regions where access to formal healthcare is limited. TBAs’ local knowledge and cultural relevance often make them trusted figures in their communities (23). Nonetheless, the challenge lies in the wide range of TBA training and experience, potentially leading to the disparities in perceptions among mothers (24). This contrast becomes even starker when considering complications, where timely medical intervention becomes crucial.

Our findings also indicated that unskilled birth attendants seemed to have built rapport with women in their communities thus creating that trust and expanding their clientele. These concur with a study conducted by Duong et al. (25) who reported that assessment of quality of services largely depended on personal experience. In essence, there was perceived trust in the manner in which birth attendants managed labor and associated outcomes which led to the perceived good quality of service. Conversely, mothers were more likely to embrace postpartum care characteristics such as satisfaction with TBA service received, courtesy and availability of newborn care kits (Mama kit).

The majority of our study participants reported satisfaction with the cleanliness of TBA delivery environments. This concurs with (26) who documented the importance of a clean birthing environment being crucial, not just for the health of the mother but also for the newborn. This may be attributed to the local understanding of cleanliness, which often differs from clinical definitions. The environment where delivery takes place plays a crucial role in the prevention of infections and complications for both the mother and the newborn (27). While many TBAs maintain hygienic practices, infrastructural challenges might limit their ability to provide ideal environments.

Cultural practices such as smearing floors with cow dung, for instance, are recognized for their significance in maintaining hygiene in some communities yet they might introduce microbial risks. On the contrary, the minority who reported dissatisfaction underscore the need for continued community education and sensitization on the importance of optimal cleanliness during childbirth.

A positive attitude and good conduct of the birth attendant during labor and delivery are essential for the psychological well-being of the mother. Most of the respondents felt they were treated well by the TBAs. This aligns with a study conducted by (28) suggesting that the interpersonal aspects of care might sometimes be better with TBAs compared to facility-based care. Yet, consistent with a study on mistreatment of women during childbirth in health facilities conducted by (29), negative experiences by some participants underline the importance of continuous training and sensitization of all birth attendants, ensuring respectful maternal care.

The behavior and attitude of healthcare providers, including TBAs, significantly impact a woman’s childbirth experience. Respectful maternity care ensures women’s dignity, privacy, and freedom from harm and mistreatment (30).

Postnatal care is critical for both the mother and newborn, providing an opportunity for health promotion, disease prevention, and early detection of problems. It is vital for monitoring complications, promoting healthy behaviors, and ensuring both the mother and infant thrive. The practice of massaging by TBAs can be traced back to traditional postnatal practices that aim at ensuring relaxation and promoting recovery. However, the integration of essential medical practices, like immunizations, into TBA care remains a challenge that needs systemic intervention (31).

Many of the participants in our study expressed satisfaction with the postnatal care provided by TBAs. These findings mirror other studies such as the 2006 meta-analysis on traditional birth attendant training and pregnancy outcomes done by (32) which found TBAs to be key in providing culturally appropriate postnatal care in some communities. Yet, the lack of essential services like immunization during the postnatal period highlights the need for a stronger linkage between TBAs and formal health care systems (33).

### Study limitations

Despite the diligent efforts to comprehensively analyze perceptions about traditional birth attendants, this study is not without its limitations and constraints. Recognizing these constraints ensures an objective assessment of the findings and aids future researchers in refining methodologies.

### Limited geographic scope

The study was conducted in a specific geographic location. Although this allowed for an in-depth analysis of the factors within this area, it also limits the study’s generalizability. The experiences and outcomes of mothers in this area may differ from those in other regions due to varying socio-cultural contexts, healthcare infrastructure, and accessibility.

### Participant selection and scope of the population

Our research concentrated on mothers availing traditional birth attendant (TBA) services, inadvertently overlooking those who might have refrained due to concerns over skill, hygiene, or other factors – a perspective that could have potentially provided intriguing contrasts. Furthermore, the study’s scope was confined to 115 mothers who gave birth outside healthcare facilities in the past 24 months. Such a selection criterion may engender bias, as it omits mothers who delivered within medical facilities or those who birthed outside them beyond the two-year frame. Such limitations could constrict the study’s breadth, potentially biasing the outcomes and affecting their wider applicability. The villages selected for the study might not represent all areas where TBAs are predominant. Thus, extrapolating the findings to a broader context requires caution.

### Self-reported data

The inherent structure of our study may raise certain concerns regarding the authenticity of the data, as it largely depends on mothers’ recollections of events. This reliance may inadvertently invite recall and social desirability biases, affecting the reliability of the data. The study’s framework may have led mothers to emphasize more memorable experiences, casting an imbalance in perceptions. Consequently, this could risk skewed or inaccurately reported experiences, potentially compromising the integrity of our findings.

### Absence of longitudinal data and direct observation

The study’s cross-sectional design prevented the collection of longitudinal data. This limitation restricts the ability to observe changes over time or to establish causality between variables. In addition, the study did not involve direct observation of TBAs during delivery. As such, perceptions of cleanliness and conduct were based solely on mothers’ accounts, without corroborative evidence.

### Implications and future directions

Despite these limitations, it is important to emphasize that the study still provides valuable insights into the experiences of mothers delivering outside healthcare facilities, which is a significant but under-researched area. The findings shed light on the reasons behind this choice, the challenges these mothers face, and the potential interventions needed. The rich qualitative data offers a foundation for future investigations and interventions aimed at optimizing the contributions of TBAs in maternal care. It is our belief that, even with its limitations, this study significantly advances the discourse on traditional birthing practices and their implications for maternal health.

The limitations discussed herein also pave the way for future research. Studies could benefit from a broader geographic scope, greater diversity in participant selection, and the use of longitudinal designs to provide a more comprehensive understanding of the issue.

In conclusion, while acknowledging these limitations, we believe that the significance of the study’s findings and their potential to inform better policies and practices in maternal healthcare should not be underestimated.

## Conclusion

Our study revealed mixed feelings among respondents regarding their perceptions and experiences with care provided during home delivery, with TBAs being assessed both positively and negatively.

Perceptions and experiences with various events and/or situations largely vary from individual to individual depending on how services were offered and the environment in which service was provided.

We established that study participants were satisfied with the hygiene of the environments upon which the TBAs conducted their deliveries. It was also reported that the conduct and attitude of TBAs was satisfactory.

With mothers’ perceived satisfaction and wide affirmation of the quality of services offered by TBAs especially during and after home deliveries, it is evident that TBAs continue to be force to reckon with in the district health system.

Our findings indicated general satisfaction with the care provided during home delivery by TBAs as expressed by mothers. Motivation to seek services from TBAs was attributable to their vast experience spanning decades with history of safe delivery. Few mothers expressed discontent with TBA services citing abuse and rudeness.

Our findings suggest that it is clear that the contributions made by the TBAs to maternal newborn and child health are significant.

We note that whereas the services offered by TBAs were abolished by the Government in 2010, the findings documented by this study on the general satisfaction with the services offered by the TBAs to mothers during and after delivery present a new dimension which should be rethought by the health authorities in the reproductive health division. Conversely, the mixed feelings toward TBAs in our study emphasize the broader debate on their role in maternal and newborn health. While they are appreciated and valued by some women, concerns around their ability to manage complications and provide comprehensive care persist. Policymakers and health professionals need to acknowledge the role TBAs play while strategizing on how to optimize maternal and newborn health outcomes in resource-limited settings. Providing them with the necessary training, and establishing clear referral pathways could be vital steps in leveraging their presence in communities while ensuring safe motherhood.

## Data availability statement

The original contributions presented in the study are included in the article/supplementary material, further inquiries can be directed to the corresponding author.

## Ethics statement

The study involved human subjects and was approved by the Makerere University, School of Public Health Higher Degrees, Research and Ethics Committee. The study was conducted in accordance with the local legislation and institutional requirements. The participants provided their informed consent to participate in this study by imprinting their thumb on the consent form.

## Author contributions

BD was responsible for conceptualization, data curation, formal analysis, investigation, methodology, resources supervision, validation, writing – original draft, and writing – review and editing. AB undertook data curation, investigation, methodology, resources validation, and writing – review and editing. JK supported data curation, investigation, resources, and writing – review and editing. BC was instrumental in crafting the methodology, validation, and writing – review and editing. All authors contributed to the article and approved the submitted version.
